# 1707. New directions for infection prevention in the neonatal intensive care unit: A key informant exploration

**DOI:** 10.1093/ofid/ofad500.1540

**Published:** 2023-11-27

**Authors:** Christopher Prestel, Laura Barnes, David Kuhar, Ronda Sinkowitz-Cochran

**Affiliations:** Centers for Disease Control and Prevention, Atlanta, Georgia; Centers for Disease Control and Prevention, Atlanta, Georgia; Centers fo Disease Control and Prevention; CDC, Atlanta, GA

## Abstract

**Background:**

Prolonged hospitalization and unique physiology of neonates and infants places them at risk for healthcare-associated infections (HAIs) in the neonatal intensive care unit (NICU). Development of infection prevention (IP) bundles led to decreases in HAIs in NICUs. We interviewed key informants to understand the IP challenges and opinions of NICU clinicians to inform unique IP needs in NICUs.

**Methods:**

Participants were referred by the Vermont Oxford Network (VON), an interdisciplinary NICU quality improvement (QI) community. A standard set of questions were used for all interviews, which covered perceived IP gaps, opportunities for QI improvement, barriers, and implementation practices. Qualitative analysis was performed using NVivo software with three key questions coded using an immersion-crystallization technique. Transcripts were systematically reviewed by a team of three coders to maintain intercoder reliability.

**Results:**

Eight subject matter experts (SMEs) were interviewed. Participants included five neonatologists, two pediatric infectious diseases physicians, and a pediatric critical care medicine physician. All participants practiced or held IP roles within a Level III or IV NICU; three were in the Northeast, three in the Midwest, and two in the Southeast region of the U.S. The most common themes to emerge across participant responses were poor hand hygiene compliance rates, inherent complexities of providing care in the neonatal population, and balancing family presence with IP concerns. The greatest opportunities for IP improvement described by SMEs were the development of novel supplemental IPC strategies to complement core practices, antibiotic stewardship in neonates, and the prevention of ventilator-associated pneumonia. The most cited barriers were a lack of evidence-based research, NICU staffing turnover and inexperience, and gaps in provider and family knowledge of IP practices.

**Conclusion:**

Identified IP challenges within the NICU are unique and cross-cutting among patient, provider, and family needs. These may be mitigated through the development of new HAI prevention tools, education, and resources developed specifically for this complex care environment.

**Disclosures:**

**All Authors**: No reported disclosures

